# Untargeted Metabolomics Study of Three Matrices: Seminal Fluid, Urine, and Serum to Search the Potential Indicators of Prostate Cancer

**DOI:** 10.3389/fmolb.2022.849966

**Published:** 2022-03-04

**Authors:** Magdalena Buszewska-Forajta, Joanna Raczak-Gutknecht, Wiktoria Struck-Lewicka, Magdalena Nizioł, Małgorzata Artymowicz, Marcin Markuszewski, Marta Kordalewska, Marcin Matuszewski, Michał J. Markuszewski

**Affiliations:** ^1^ Institute of Veterinary Medicine, Faculty of Biological and Veterinary Sciences, Nicolaus Copernicus University in Toruń, Torun, Poland; ^2^ Department of Biopharmaceutics and Pharmacodynamics, Faculty of Pharmacy, Medical University of Gdańsk, Gdańsk, Poland; ^3^ Department of Pharmaceutical and Biopharmaceutical Analysis, Faculty of Pharmacy, Medical University of Białystok, Białystok, Poland; ^4^ Department of Urology, Faculty of Medicine, Medical University of Gdańsk, Gdańsk, Poland

**Keywords:** prostate cancer, seminal fluid, urine, plasma, metabolomics, metabolites, HPLC

## Abstract

The simultaneous determination of metabolites from biological fluids may provide more accurate information about the current body condition. So far, the metabolomics approach has been successfully applied to study the mechanism of several disorders and to search for novel biomarkers. Urine and plasma are widely accepted matrices for the evaluation of several pathologies, while prostate cancer (CaP) development is still unknown. For this reason, an alternative matrix, the seminal fluid, was proposed to expand the knowledge about the CaP pathomechanism. The main aim of this study was to develop and optimize the sample preparation protocol to ensure the highest coverage of the metabolome of ejaculate samples. Parameters like the type and composition of the solvent mixture, time of extraction, and applied volume of the solvent were tested. The optimized method was applied for the untargeted metabolomics profiling of seminal fluid samples obtained from CaP patients. Moreover, urine and serum samples were also prepared for untargeted metabolomics analysis. Analyses were carried out with the use of two complementary analytical techniques: GC-EI-QqQ/MS and LC-ESI-TOF/MS. Finally, the metabolic signature of seminal fluid (n = 7), urine (n = 7), and plasma (n = 7) samples was compared. Furthermore, the hypothesis of the increased level of metabolites in ejaculate samples related to the CaP development was evaluated. The results indicated that the developed and optimized sample preparation protocol for seminal fluid may be successfully applied for metabolomics study. Untargeted analysis of ejaculate enabled to determine the following classes of compounds: fatty acids, sphingolipids, phospholipids, sugars, and their derivatives, as well as amino acids. Finally, a comparison of the three tested matrices was carried out. To our best knowledge, it is the first time when the metabolic profile of the three matrices, namely, urine, plasma, and seminal fluid, was compared. Based on the results, it can be pointed out that ejaculate comprises the metabolic signature of both matrices (polar compounds characteristic for urine, and non-polar ones present in plasma samples). Compared to plasma, semen samples revealed to have a similar profile; however, determined levels of metabolites were lower in case of ejaculate. In case of urine samples, compared to semen metabolic profiles, the levels of detected metabolites were decreased in the latter ones.

## 1 Introduction

Cancer is one of the most common causes of death worldwide, and the death rate increases with the age of the population, particularly in less developed countries. The unhealthy lifestyle behaviors, such as smoking, bad diet, low physical activity, and reproductive changes, increase the risk of cancer (Torre et al., 2012; Labbé et al., 2019). Prostate cancer (CaP) is the most common age-related cancer in men worldwide, which includes about 1,600,000 cases and 366,000 deaths annually (Torre et al., 2012). Despite recent medical progress, CaP remains a significant problem in men (Torre et al., 2012; Etheridge et al., 2018; Maggi et al., 2019). At an early stage, CaP is usually asymptomatic, which leads to late diagnosis, when the patient’s prognosis is poor. Most often, it is detected during screening serum prostate-specific antigen (PSA) tests. PSA is regarded as the standard diagnostic CaP marker. However, the level of PSA is not cancer-specific and can be increased due to other processes such as prostate hypertrophy and inflammation (Van Gils et al., 2007; Etheridge et al., 2018; Bruno et al., 2021; Cacciatore et al., 2021).

Men with significantly elevated PSAs and other criteria (age, cancer in the family line, and results from digital rectal examination) are subjected to the prostate biopsy, which may cause side effects and often leads to false-negative results. Some patients avoid this examination because of discomfort connected with the procedure. Therefore, there is an urgent need for finding non-invasive, sensitive, and specific tests which could identify clinically significant CaP cases and reduce the number of unnecessary prostate biopsies (Busetto et al., 2021; de la Calle et al., 2021; Maggi et al., 2021).

The interesting body fluid which may be helpful to find new potential CaP biomarkers is ejaculate. Many studies have been conducted with the use of plasma or urine, while only a few have been focused on the analysis of seminal fluid (Van Gils et al., 2007; Cerrato et al., 2021; Salciccia et al., 2021). In addition to sperms, which account for about 1% of ejaculate composition, seminal fluid also contains spermine and spermidine—compounds from the group of polyamines, cadaverine—responsible for the smell and taste of sperms, ascorbic acid, citrate, prostaglandins, enzymes, amino acids, steroid hormones, cobalamin, fructose, urea, cholesterol, and minerals (potassium, zinc, and calcium) (Ganong, 1997). Analyses of exosomes, proteomes or metabolomes may accelerate and improve diagnostics of the disease (Logozzi et al., 2020; Logozzi et al., 2021; Salciccia et al., 2021). In previous studies, it was (Goessl et al., 2000) shown that molecular and metabolomic markers in the ejaculate or seminal fluid have been reported to improve diagnosis compared to (Tinzl et al., 2004) serum PSA (Neuhaus et al., 2013; Selth et al., 2014).

Glutathione S-transferase Pi 1 (GSTP1), found as CaP biomarkers, can be detected in semen ejaculate (Bruno et al., 2021). Also, CaP antigen 3 (PCA3) was detected from shed CaP cells in prostatic fluid and urine. The reported sensitivity and specificity of the seminal fluid PCA3 test and urine PCA3 test suggested that seminal fluid is a better source of this protein than urine (Van Gils et al., 2007; Etheridge et al., 2018; Cacciatore et al., 2021). Based on this, it can be assumed that the study based on ejaculate samples may provide new insights into CaP development. Human seminal ejaculate is composed of sperms and secretions from glands in the male urogenital tract. Approximately, 40% is material originating from the prostate (Roberts et al., 2016). It contains exocrine and constituents of the prostate, such as PCa-derived cells, proteins, and metabolites, or PCa-associated miRNAs (Selth et al., 2014). Prostatic constituents are highly concentrated than other body fluids. PSA was originally found in seminal fluid, where it exists at about 5-6 times higher concentration than in blood serum (Lilja, 1985). Malignant prostatic epithelial cells and their products can only enter the circulation following transgression of blood–tissue barriers, while normal and cancer glands release their secretions naturally into seminal fluid (Roberts et al., 2015). For this reason, it can be assumed that the level of some metabolites may be higher in ejaculate than in other biological matrices. From the clinical point of view, some biomarkers could be detected earlier in seminal fluid than in blood, at an early stage of the disease. Seminal fluid is supposed to contain cancer cells. Therefore, changes in metabolic profiles can be detected much faster in semen samples than in traditional histopathological examination samples.

Moreover, the levels of metabolic biomarkers in blood and urine can be disturbed by high variability due to physical and metabolic processes in contrast to the seminal fluid. Also, postejaculatory urine may be an attractive biofluid that contains both prostate-derived and urine-specific biomarkers. However, key biomarkers are present at lower concentrations in postejaculatory urinethan in seminal fluid. Therefore, highly sensitive instrumentation would be required to detect such signals.

Recently, the attention to seminal plasma has also been highlighted. There is increasing evidence that seminal plasma might be the original source of potential markers for prostatic malignancy (Obiezu et al., 2005; Schiffer, 2007).

The main aim of this study was to develop and optimize an efficient method of extracting low-molecular weight compounds from human sperms suitable for metabolomics study. Moreover, untargeted metabolomics profiling of biological samples obtained from patients with diagnosed CaP was performed. For this purpose, three types of matrices were analyzed, namely, ejaculate, serum, and urine. The samples were analyzed with the use of two complementary analytical platforms, namely, high-performance liquid chromatography coupled to mass spectrometry (HPLC-ESI-TOF/MS) and gas chromatography coupled with mass spectrometry (GC-EI-QqQ/MS).

To the best of the authors’ knowledge, this is the first study where ejaculate samples were analyzed using the metabolomics approach and compared with two other matrices. The hypothesis about the increased level of metabolites in ejaculate samples which can be related to CaP development was evaluated. For this reason, the development and optimization of the new sample preparation method of ejaculate samples were carried out. Twelve sample preparation protocols were tested in order to obtain both the most reliable sample preparation procedure and most complex metabolomic profile of seminal fluid.

## 2 Experimental

### 2.1 Chemicals

All chemicals used were of HPLC-MS or analytical grade. Urease was obtained from Sigma-Aldrich (St. Louis, MO, United States). Pentadecanoic acid, methoxyamine hydrochloride, pyridine, and *bis-N-O* trimethylsilyl trifluoroacetamide (BSTFA) were purchased from Sigma-Aldrich (St. Louis, MO, United States). Chlorotrimethylsilane (TMCS) was provided by Supelco (Supelco, United States). Heptane, ethanol, and methanol were provided by J.T. Baker (Arnhem, Netherlands). Ultrapure water was obtained using a Milli-Q Plus water system from Millipore (Zug, Switzerland).

2.2 Sample collection of biological material

### 2.2. Study Subjects

All the participants (n = 7) enrolled in the study were diagnosed with CaP. Each patient was qualified for radical prostatectomy on the basis of examination carried out at the Department of Urology, Medical University of Gdańsk. The mean age of the participants was 62 ± 13 years. Each cancer case was diagnosed according to the Gleason grading score and Grade Groups WHO 2016, and pTNM was assessed (Penney et al., 2021). The characteristics are listed in Table 1. Each patient treated with hormones and with diagnosed metastatic organs, diabetes, or any other chronic diseases was excluded from the study. The studies were performed in accordance with the principles embodied in the Declaration of Helsinki and executed according to the Independent Commission for Bioethics Research, Medical University of Gdańsk (number of constents NKBBN/432—119/2017 and NKBBN/433/2016). All the subjects gave a written consent to participate. From each patient, three types of biological materials, namely, ejaculate, serum, and urine, were collected and stored at −80°C up to the day of analysis. Directly before analysis, biological samples were thawed at room temperature.

**TABLE 1:** Clinical and pathological characteristics of the population.

Type of biological materials	Urine, plasma, and semen
Number of cap samples	7
Mean age (years)	62 ± 13
Gleason score (gs)	
GS = 7	7(100)
3 + 4 =7	
Pathological state (%)	
Stage 2	100
Psa level (ng ml^−1^)	
Range	4.03–25.381

Age [years], PSA level: prostate-specific antigen [ng/mL]; GS: Gleason score indicates the degree of tumor (letters indicate the type of classification scale, and number corresponds to degree of tumor malignancy), Pathological state indicates that cancer cells were identified only in prostate gland.

### 2.3 Sample Preparation Procedure

#### 2.3.1 Optimization for Extraction Procedure of Semen Samples

The optimization of the sample preparation protocol covers the type of solvent, volume of solvent, and time of incubation. In case of the type of the solvent, three types of extraction solutions, namely, pure methanol, pure acetone, and a mixture composed of methanol and ethanol (1:1, *v/v*), were tested. Two volumes of solvent, namely, 300 and 600 µl, and two incubation times (15 and 30 min) were taken into account. Finally, twelve extraction protocols were studied with the use of representative sample, obtained by the pooling of semen samples (n = 7). Three replications were generated for each tested method. Each sample contained 15 mg of seminal fluid. All of the tested methods are presented in Table 2.

**TABLE 2:** Analytical characteristics of the developed methods.

Method	Extraction solvent	Volume of solvent [µL]	Extraction time [min]
*1*	Methanol/ethanol (1:1, *v/v*)	300	15
*2*	Methanol/ethanol (1:1, *v/v*)	300	30
*3*	Methanol/ethanol (1:1, *v/v*)	600	15
*4*	Methanol/ethanol (1:1, *v/v*)	600	30
*5*	Methanol	300	15
*6*	Methanol	300	30
*7*	Methanol	600	15
*8*	Methanol	600	30
*9*	Acetone	300	15
*10*	Acetone	300	30
*11*	Acetone	600	15
*12*	Acetone	600	30

The efficiency of the deproteinization process was improved by incubating at −20°C. The samples were analyzed using the LC-TOF/MS technique described in Section 2.4.

#### 2.3.2 Sample Extraction Procedure for LC-MS

##### Extraction Procedure for Urine

Initially, urine samples were vortex-mixed for 1 min, 200 µl of the urine was transferred to a new probe, and 200 µl of deionized water was added to it. The prepared samples were centrifuged (4,000 × g for 15 min) and filtered directly into HPLC vials using 0.22-μm nylon filters (Agilent Technologies, Waldbronn, Germany).

##### Extraction Procedure for Serum Samples

Each serum sample was vortex-mixed, and 50 µl of the serum was transferred to a new Eppendorf tube. Then, 150 µl of a cold mixture of methanol and ethanol (1:1, *v/v*) was added, vortex-mixed for 5 min, and stored at −20°C for deproteinization. Finally, the samples were centrifuged for 15 min (13,000 rpm, 4°C). The supernatant was collected, and the extracts were filtered and collected in HPLC vials using nylon filters (d = 0.22 µm; Agilent Technologies, Waldbronn, Germany).

##### Extraction Procedure for Semen Samples

Each semen sample containing 15 mg of semen was homogenized with 300 µl of cold methanol. The homogenization process was carried out using a Bullet Blender Storm Homogenizer (Next Advance, United States). Homogenization was carried out in three cycles, each composed of a homogenization step (1 min) and cooling in the ice (1 min). Finally, the homogenized samples were placed at −20°C for 15 min. Then, the samples were centrifuged for 15 min (13,000 rpm, 4°C), and supernatants were collected. Finally, the extracts were filtered into HPLC vials with the use of 0.22-µm nylon filters (Agilent Technologies, Waldbronn, Germany).

#### 2.3.3 Extraction Protocol for GC–MS

##### Extraction Procedure for Urine

In the first step, 50 μl of urease solution (Sigma-Aldrich, United States, 600 units/ml; 0.0085 g/ml in Milli-Q water) was added to 200 μl of urine and vortex-mixed for 1 min. Then, the samples were incubated at 37°C for 30 min in order to decompose and remove the excess amount of urea (Webb-Robertson et al., 2014). Then, 10 µl of *n*-pentadecanoic acid (IS) in methanol (Sigma-Aldrich, United States, 1 mg/ml in methanol) and 800 µl of cold methanol were added to the samples. The samples were then centrifuged for 15 min (13,000 rpm, 4°C); 200 µl of pure supernatant was transferred to a new glass insert and evaporated to dryness.

##### Extraction Procedure for Serum Samples

In brief, 50 µL of each collected serum was transferred to a new Eppendorf tube. In the first step, 5 µl of *n*-pentadecanoic acid in methanol and 150 µl of cold methanol were added. The samples were vortex-mixed at room temperature for 5 min and stored at −20°C for 10 min to increase the efficiency of deproteinization. Then, samples were centrifuged for 15 min (13,000 rpm, 4°C); 100 µL of pure supernatant was transferred to a glass insert. The next step was evaporation, which was carried out for 2 h at 36°C with the use of a Quatro-Vac (GeneVac, Great Britain). The dry residue was dissolved in derivatization agents.

##### Extraction Procedure for Semen Samples

Each 15 mg of semen was transferred into a separate tube. Then, 300 µl of cold methanol was added, and the samples were homogenized using a Bullet Blender Storm Homogenizer (Next Advance, United States). The homogenization process was carried out with the use of three-cycle procedure, each cycle has a homogenization step (1 min) and cooling in the ice (1 min). Finally, the homogenized samples were placed at −20°C for 15 min in order to improve the efficiency of deproteinization. Then, the samples were centrifuged for 15 min (13,000 rpm, 4°C), and supernatants were collected. Similar to urine and serum samples, the supernatant of seminal origin was evaporated for 2 h at 36°C with the use of a Quatro-Vac (GeneVac, Great Britain). Finally, the two-step derivatization process was carried out.

##### Derivatization Process

The protocol of the derivatization process was the same for each out of three analyzed matrices evaporated to dryness. In brief, in the first step, methoxymation was carried out. For this purpose, the dry residue was dissolved in 30 μl of methoxyamine (Sigma-Aldrich, Switzerland) in pyridine (Sigma-Aldrich, Germany) in a concentration of 15 mg/ml and vortex-mixed for 10 min. The samples were stored for 16 h at room temperature in a dark place. Second, the silylation process was followed by the addition of 30 μl of BSTFA with 1% TMCS. The samples after vortex-mixing were incubated for 1 h at 70°C. Finally, the samples were diluted with the use of 100 μl of heptane (J.T. Baker, Great Britain) and vortex-mixed for 10 min. Subsequently, 1 µl of the sample was introduced into the GC–MS system. Blanks comprised the pure solvent heptane in which the final extract was diluted and was included in each sample sequence.

#### 2.3.4 Quality Control Sample Preparation

Quality control (QC) samples were prepared by mixing the same volume of each of the analyzed samples within a given matrix. In case of urine, the pooled sample was obtained by mixing 100 μl of each of the 7 urine samples, while a representative serum sample was obtained by the pooling of 50 μl of each of the 7 serum samples. The pooled semen sample was obtained by the mixing of 5 mg of each collected semen sample. The pooled sample was divided into two QC samples, one for each applied technique. Each the QC sample contained 15 mg of pooled semen.

The QC samples were prepared for two applied techniques. For HPLC, separate tubes with 50 μL of pooled serum sample, 200 μL of pooled urine sample, and 15 mg of pooled semen sample were prepared and proceeded with the same protocol as regular samples analyzed using the HPLC-TOF/MS method (Section 2.3.2). The QC samples were injected at the beginning of the assay and after every 2 samples.

Similarly, the QC samples were prepared for GC–MS analysis. In brief, 2 samples (each 50 µl of serum from the mixture, each 200 μl of pooled urine sample, and each 15 mg of pooled semen sample) were prepared in accordance with the protocol described in Section 2.3.2. The QC samples were injected at the beginning of the sequence run and after every 2 regular samples.

The analyses of the QC samples allowed monitoring the stability of the analytical system and reproducibility of analytical methods used. Blank samples, with adequate amount of organic fraction (50 µl for serum and 200 µl for urine), were processed with the use of the same pretreatment procedure as in the case of biological samples.

### 2.4 Metabolic Fingerprinting With LC-ESI-TOF/MS

Metabolomics analysis was performed with the use of 1260 HPLC coupled with the 6224 TOF/MS system (Agilent Technologies, Waldronn, Germany) equipped with an electrospray ionization (ESI) source. Before analysis, the system was calibrated with the use of low-concentration tuning mix, dedicated ESI-L calibration solution (Agilent Technologies, Germany). The mobile phase composed of 0.1% aqueous solution of formic acid (A) and 0.1% formic acid in acetonitrile (B), with a flow rate of 0.35 ml/min. Analyses were carried out in a gradient elution mode as follows: starting with 2% of B and increased to 98% within 25 min. Then, the mobile phase with composition 2% of A and 98% of B was set unchanged within 10 min. The equilibration time was set to 10 min. Chromatographic separation of 1 μl of each sample was carried out using the Ascentis Express-C18 reversed-phase (RP) column (4.6 mm × 1,500 mm, 2.7 μm; Bellefonte, PA, United States) within 35 min. The MS parameters at the optimized conditions were as follows: gas flow 10 L min^−1^, nebulizer 30 psig, capillary voltage 3250 V, fragmentor voltage 150 V, drying gas temperature 325°C, and skimmer voltage 65 V. Analyses were carried out in a scan mode, and spectra were registered in the mass range from 50 to 1,100 *m/z*, in a positive mode. Data acquisition and processing were performed using MassHunter software version B.04.00 (Agilent Technologies, Walbronn, Germany).

**FIGURE 1:**
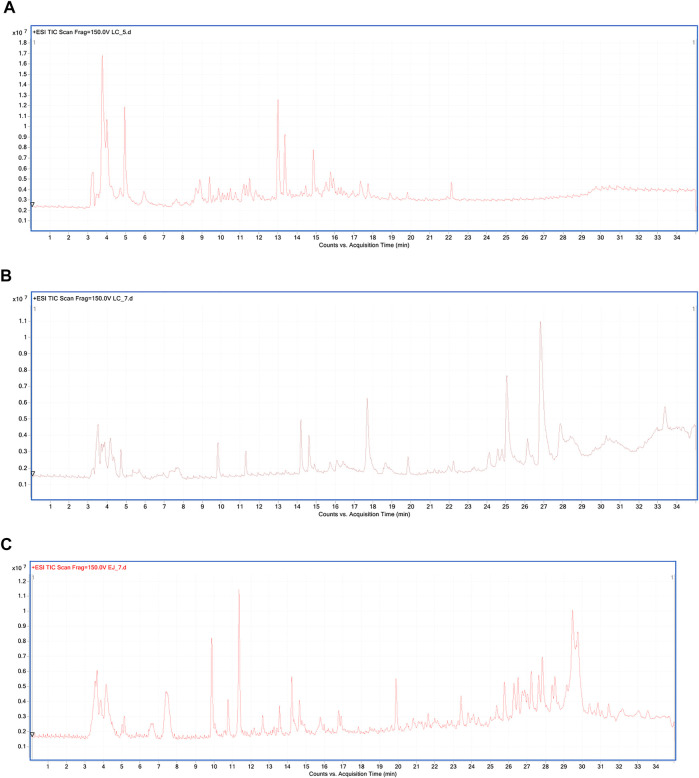
Representative total ion chromatogram (TIC) of urine **(A)**, serum **(B)**, and semen **(C)** metabolic fingerprints obtained with LC-ESI-TOF/MS analysis in the positive ionization mode.

The representative total ion chromatograms of urine, serum, and semen metabolic fingerprints obtained using LC-ESI-TOF/MS analysis in a positive ionization mode are displayed in Figure 1.

### 2.5 GC–MS Analysis

Fingerprinting analyses of samples were carried out with the use of a GC-QqQ/MS 8030 System (Shimadzu, Japan). In total, 1 µl of prepared samples was injected into the Zebron ZB-5MS (30 m × 0.25 mm i. d., 0.25 μm film thickness) capillary CG column (Phenomenex, Torrance, CA, United States) in the splitless injection mode.

Analyses were carried out in the following gradient temperature program: 0–1 min, 60°C; 1–36 min, 60–320°C; and 36–41 min, 320°C. Samples were injected in the splitless injection mode. The temperature of the injector, ion source, and interface temperature was set as follows: 250, 200, and 300°C, respectively. The analysis was carried out in the positive electron impact ionization mass spectrometer, with an ionization voltage of 70 EV in a scan range from 50 to 600 m*/*z. The system was calibrated with the use of perfluorotributylamine (PFTBA). At the beginning of the sequence, the analysis of even alkanes’ mixture (C10 to C40) was conducted. For this purpose, 2 drops of stock solution of alkanes (50 mg/L in heptane; Sigma-Aldrich, United States) were diluted in 900 µL of heptane. The obtained data were used to calculate the retention index, on the basis of which the determined metabolites were preliminary identified.

**FIGURE 2:**
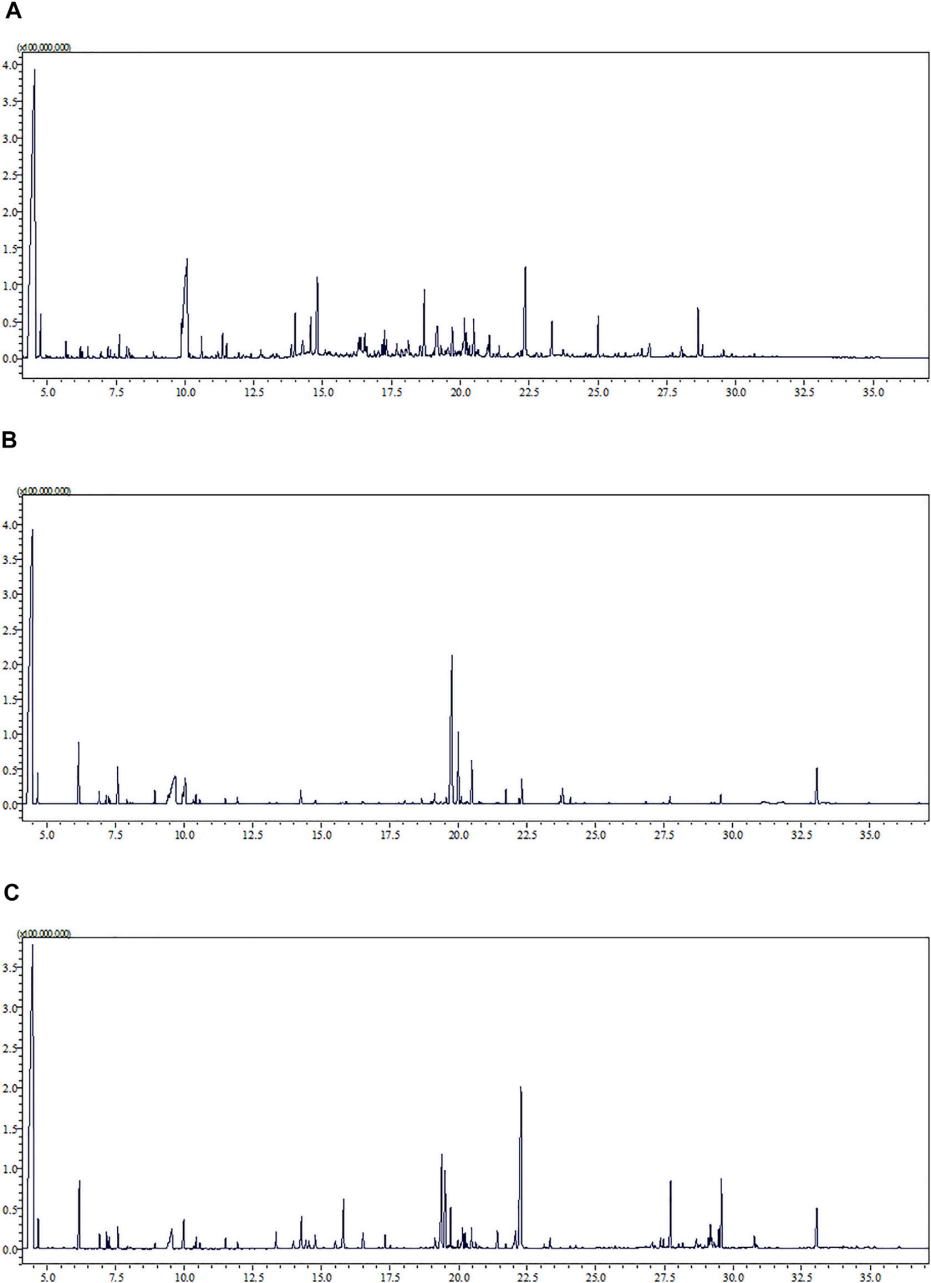
Representative total ion chromatogram (TIC) of urine **(A)**, serum **(B)**, and semen **(C)** metabolic fingerprints obtained with GC–MS analysis.

The representative total ion chromatograms of urine (A), serum (B), and semen (C) metabolic fingerprints obtained with GC–MS analysis are presented in Figure 2.

## 3 Data Treatment, Statistical Analysis, and Metabolite Identification

### 3.1 LC-MS Data

First of all, deconvolution of raw datasets was performed with the use of MassHunter Qualitative Analysis software (B.06.00, Agilent Technologies, Waldbronn, Germany). The obtained dataset included information regarding monoisotopic mass, retention time, and abundance. In the next step, data alignment was carried out using Mass Profiler Professional software (B.02.01., Agilent Technologies, Waldbronn, Germany), where parameters such as retention time correction and mass correction were set to 1.0% and 5 ppm, respectively. The aligned datasets were filtered regarding both quality assurance (QA) (Cerrato et al., 2021) criteria and presence in the compared groups. Only variables present in at least 50% of QCs and representing a coefficient of variation (CV) lower than 20% for LC-ESI-TOF/MS present in at least 85% of samples (6 out of 7 samples) were proceeded for normalization.

In case of LC-ESI-TOF/MS, normalization based on the MSGUS (MS Group Useful Signal) algorithm was applied, where the normalization factor is an average value of intensities for each compound present in 100% of analyzed samples. The obtained datasets were subjected to statistical analysis. The normality of data distribution was checked with the use of Shapiro–Wilk test, while homogeneity of variance between groups was evaluated using Levene’s test. Finally, ANOVA was performed to discover the differences between the analyzed types of matrices, while post hoc tests were performed to explain which metabolites differed statistically significantly. ANOVA and the post hoc test were performed with the use of Metaboanalyst, 4.0 software; PCA was performed using Simca P+ 13.0.3 software (Umetrics, Umea, Sweden). The results were subjected to the false discovery rate (FDR) algorithm in order to minimize the risk with false-positive results. The statistically significant variables were putatively identified based on monoisotopic mass, isotopic distribution, formula, and hits found in publicly available databases such as METLIN (www.metlin.scripps.edu), KEGG (www.genome.jp/kegg), LIPIDMAPS (www.lipidmaps.org/), HMDB (www.hmdb.ca), and CEU MassMediator (http://ceumass.eps.uspceu.es/mediator).

### 3.2 GC–MS Data

Total ion chromatograms were extracted with the use of Mass GC/MS Solution Software version 4.01 (Shimadzu, Kyoto, Japan). The obtained dataset was deconvoluted using Automated Mass Spectral Deconvolution and Identification Software (AMDIS). Identification was carried out on the basis of the retention index, retention time, and spectra included in the NIST 14 library. In brief, in the first step, the retention indices for all detected metabolites were calculated in reference to known retention indices for analyzed alkanes. Second, normalization of retention time for the compound was performed by the retention time and retention index of nearest eluting alkane. Finally, data alignment and filtration were performed. Only metabolites present in 50% of QC samples, with a coefficient of variation (CV) lower than 30% and present in 85% of samples, were selected for further investigation. Afterward, the obtained dataset was normalized by the intensity of pentadecanoic acid and selected as an internal standard. In case of semen samples, normalization by the weight of samples was also carried out. The multivariate statistical comparisons were performed with the use of Metaboanalyst, 4.0 and Simca P+ 13.0.3 software (Umetrics, Umea, Sweden). The identification was carried out on the basis on RI, RT, and NIST 14 and in-house spectral libraries.

## 4 Results

### 4.1 Development of Sample Extraction Protocol for Semen Samples

**FIGURE 3:**
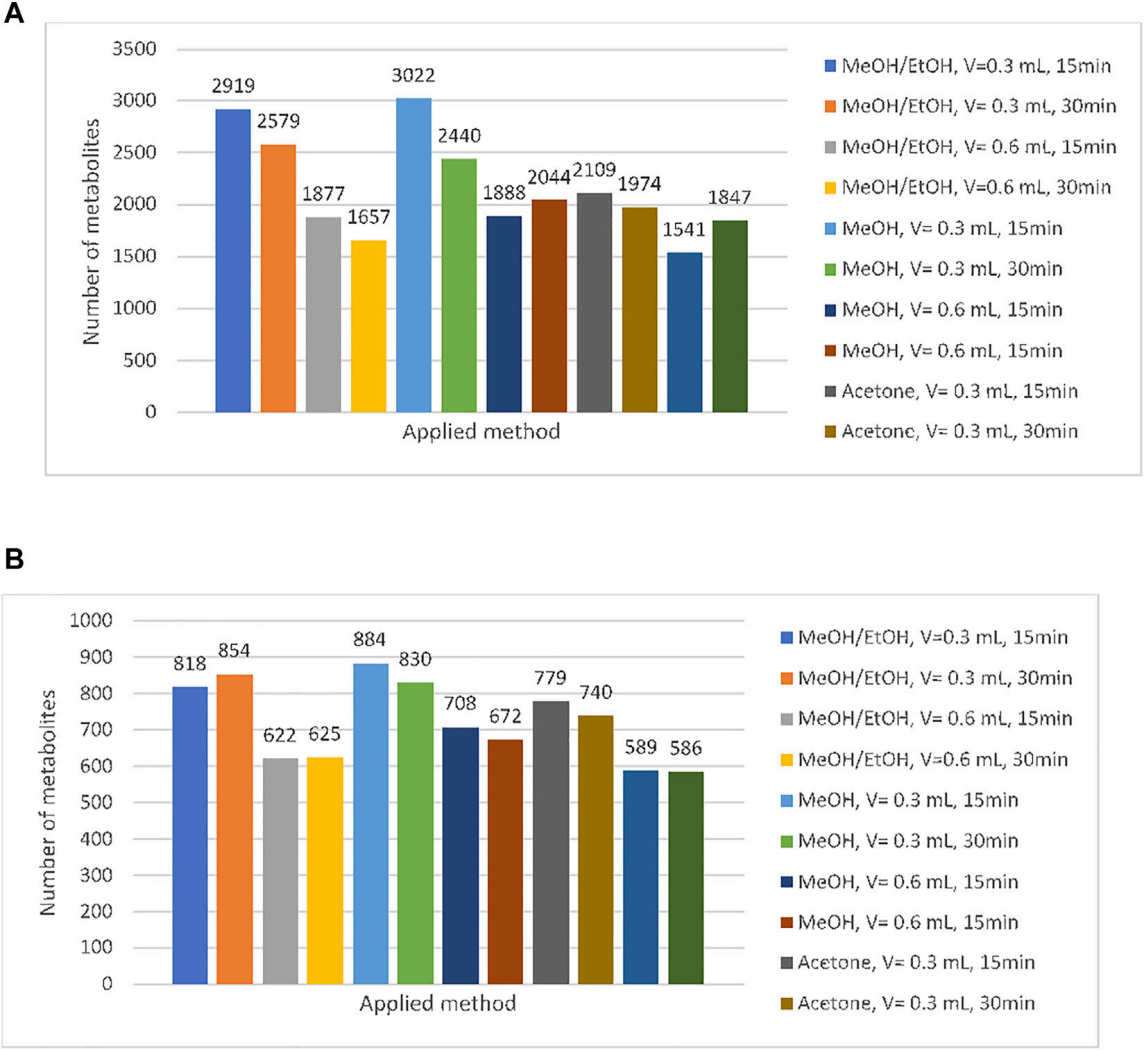
Number of compounds in reference to applied method (CV < 30%) **(A)** and number of compounds regarding the presence in all analyzed replication and developed method **(B)**.

Twelve methods were developed in order to select one that made it possible to obtain the richest and the most reproducible metabolic profiles of the ejaculate. For this purpose, three factors, namely, composition of extraction mixture, time of incubation, and the volume of solvent, were taken into account. In accordance with the composition of the extraction mixture, three types of solvents were used: methanol/ethanol (methods 1–4), methanol (methods 5–8), and acetone (9–12). For each type of the extraction solvent, two volumes, namely, 300 and 600 µl, were tested. Moreover, the influence of the time of incubation (15 and 30 min) was checked. A detailed description of the tested methods is presented in Table 1 (Section 2.3.1). The obtained dataset was filtered in accordance with QC and QA criteria. First of all, we selected metabolites which were determined in each repetition for each out of tested sample preparation method. The obtained results are listed in Figure 3A. Second, we selected metabolites with a coefficient of variation less than 30% (CV<30%) for each of the tested sample preparation methods. CV was calculated in reference to all replications. The results are shown in Figure 3B.

As can be observed, the application of pure methanol as an extraction solvent enables to determine the highest number of variables. Methods based on extraction with methanol (5–8) can be characterized by the highest repeatability (CV<30%), while utilization of acetone (methods 9–12) seems to be the most resistant to the other tested factors (time of incubation and the volume of solvent). For this reason, methanol was selected as the most suitable extraction medium for semen samples.

**FIGURE 4:**
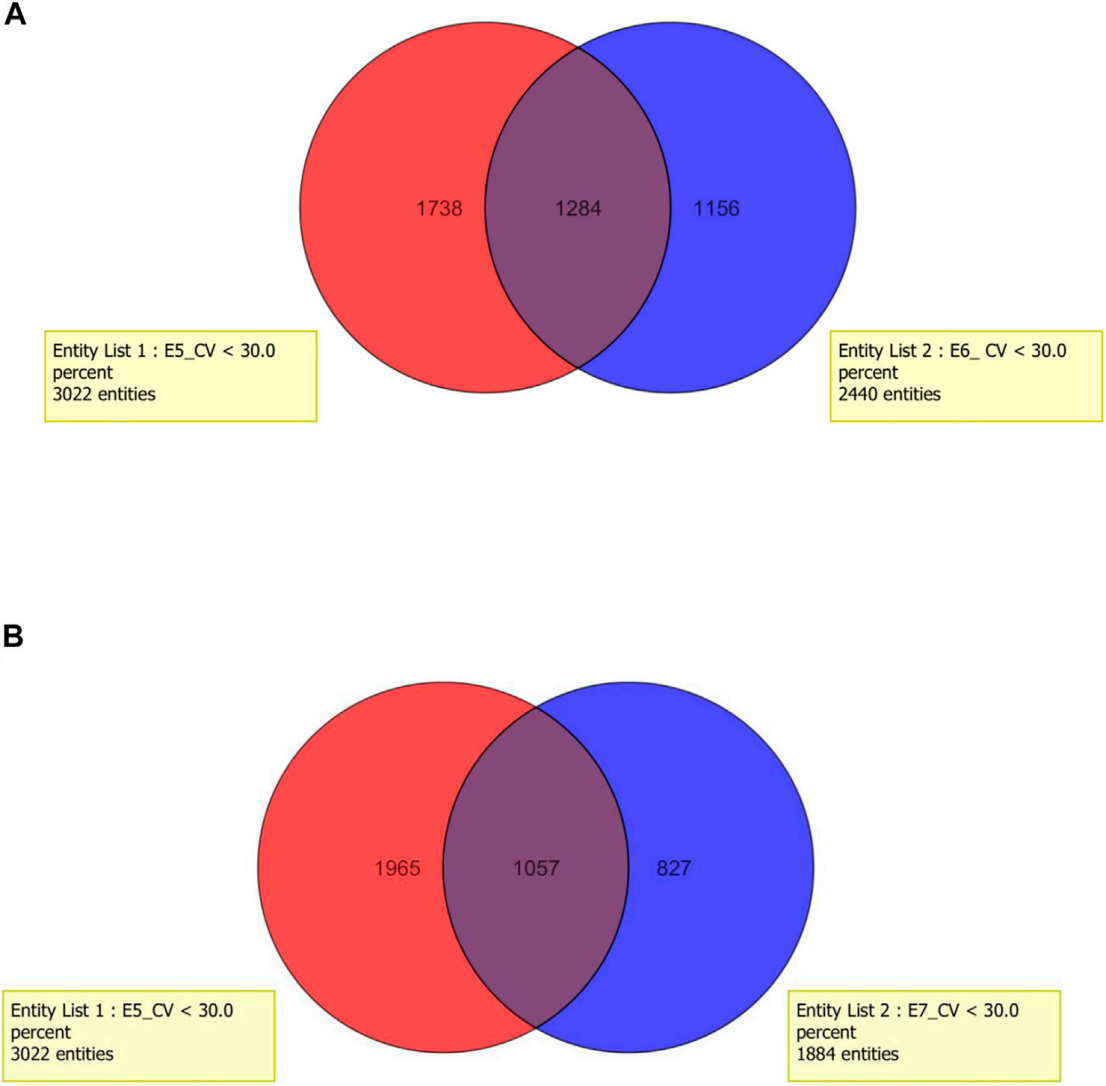
Venn diagram for two tested factors: **(A)** time of incubation within deproteinization process and **(B)** volume of extraction mixture.

In the next step, the time of incubation (15 min or 30 min) was taken into account. As it can be observed, in each of the tested extraction solvent, higher efficiency of deproteinization was obtained in the 15-min incubation (methods: 1, 3, 5, 7, 9, 11). Considering the results obtained with the methanolic extract, 3,022 metabolites were determined in samples incubated within 15 min (SD < 30%), while 30-min incubation enabled to determine 2,440 metabolites. A detailed analysis regarding the composition of methanolic extracts shows that 1,284 metabolites were determined using both extraction protocols, 1738 were released with the use of method 5, and 1,156 entities were specific for method 6. Similarly, observation was made regarding the volume of solvent. Higher repeatability and richer profiles were obtained when 300 μL was used for sample homogenization (methods: 1, 3, 5, 6, 9, and 10). The application of higher volume of extraction solvents (600 μL which corresponds to method number: 2, 4, 6, 8, 10, and 12) provided reduction of detected variables at about 38%. On the basis of methanolic extracts, it can be observed that extraction carried out with the use of 300 μL of methanol enables to determine 3,022 variables, while using 600 μL of this solvent leads to a decrease in the number to 1,884. Among them, 1,057 variables were determined with the use of both tested methods. Results are presented in the form of Venn diagrams in Figure 4.

The choice of the method was carried out on the basis of two parameters: the number of determined analytes (presence in 100% of analyzed samples, n = 3) and reproducibility (coefficient of variation (CV) was lower than 30%) in obtaining the metabolic profiles of the ejaculate.

According to the obtained results, a method utilizing 300 µl of methanol was used as the extraction solvent, and 15-min incubation time was applied. This method demonstrates the best extraction ability and was selected for further studies.

### 4.1 Determination of Metabolomics Profile of Ejaculate Samples

Method development and optimization enabled to select the most appropriate method for untargeted metabolomics analysis. Its application provides information about the metabolomics profile of ejaculate samples. Identification was carried out based on monoisotopic mass, isotopic distribution, formula, and hits found in available databases. For this purpose, selected analytical signals were putatively identified with such databases as CEU MassMediator (http://ceumass.eps.uspceu.es/mediator), LIPIDMAPS (www.lipidmaps.org/), HMDB (www.hmdb.ca) KEGG (www.genome.jp/kegg), and METLIN (www.metlin.scripps.edu).

**FIGURE 5:**
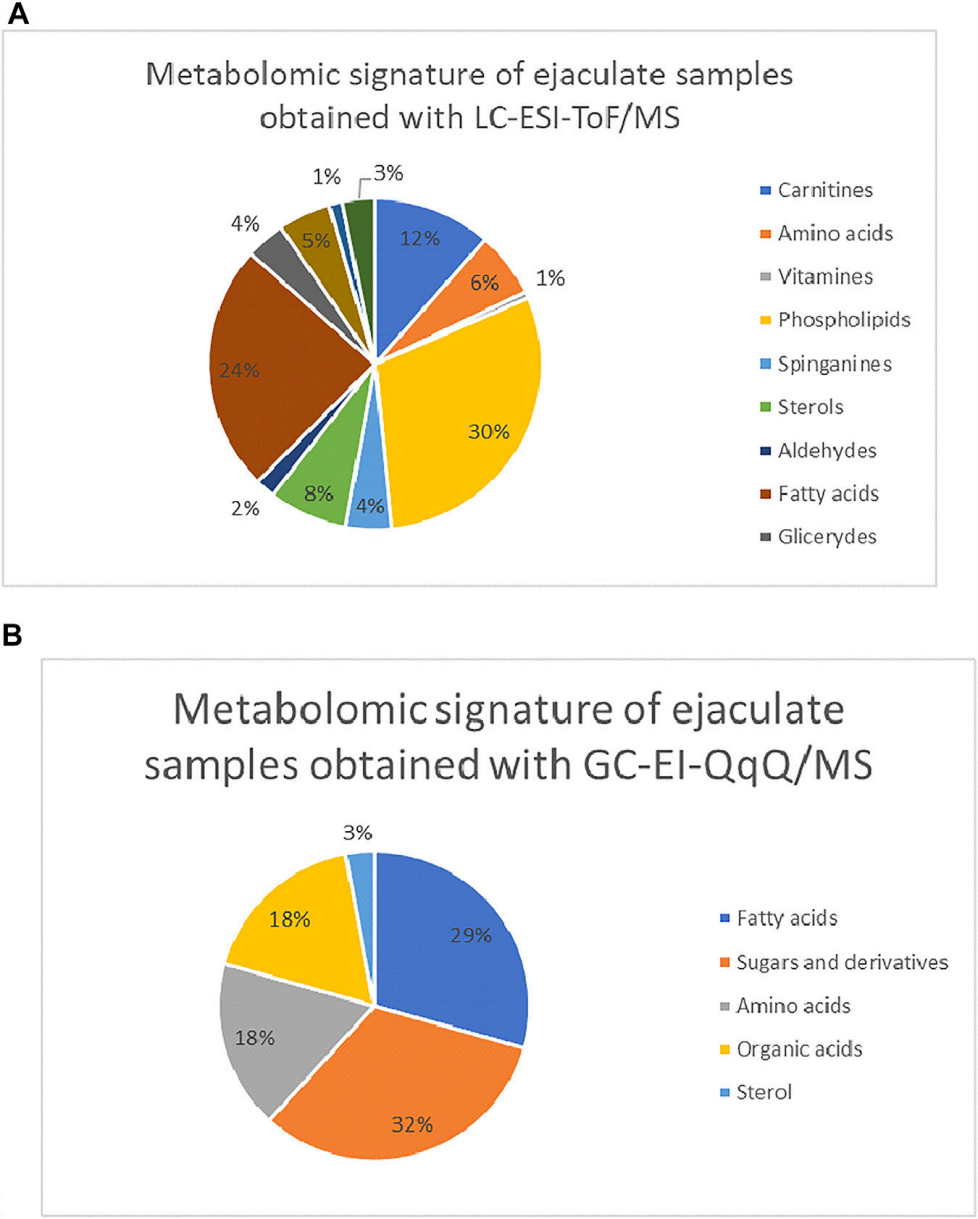
Composition of metabolomics signature of ejaculate samples divided into the particular groups of compounds **(A)** for the LC-ESI-TOF/MS study and **(B)** GC-EI-QqQ/MS. 5.1 statistical comparisons.

Finally, 124 variables were putatively identified with the use of LC-ESI-TOF/MS. Among them, phospholipids (30%), carnitines (12%), amino acids (6%), fatty acids (24%), sterols (3%), sphinganines (4%), and glycerides (4%) were annotated. The most numerous group of compounds was phospholipids and fatty acids, which proved that ejaculate is a fatty compound-rich matrix. Similar results were obtained with the use of a second analytical approach, GC-EI-QQQ/MS. The application of gas chromatography enabled to determine 37 compounds which are divided into the following classes: fatty acids, amino acids, one sterol, and sugar and its derivatives. The percentage share of individual classes of compounds in the metabolic profile of ejaculate samples is presented in Figure 5. Moreover, a detailed list of putatively identified metabolites in ejaculate samples with the use of the LC-ESI-TOF/MS technique is given in Table 1SM included in the supplementary material, while compounds determined with the use of the GC-EI-QqQ/MS approach are presented in Table 2 of the supplementary material.

#### 4.1.1 Principal Component Analysis

**FIGURE 6:**
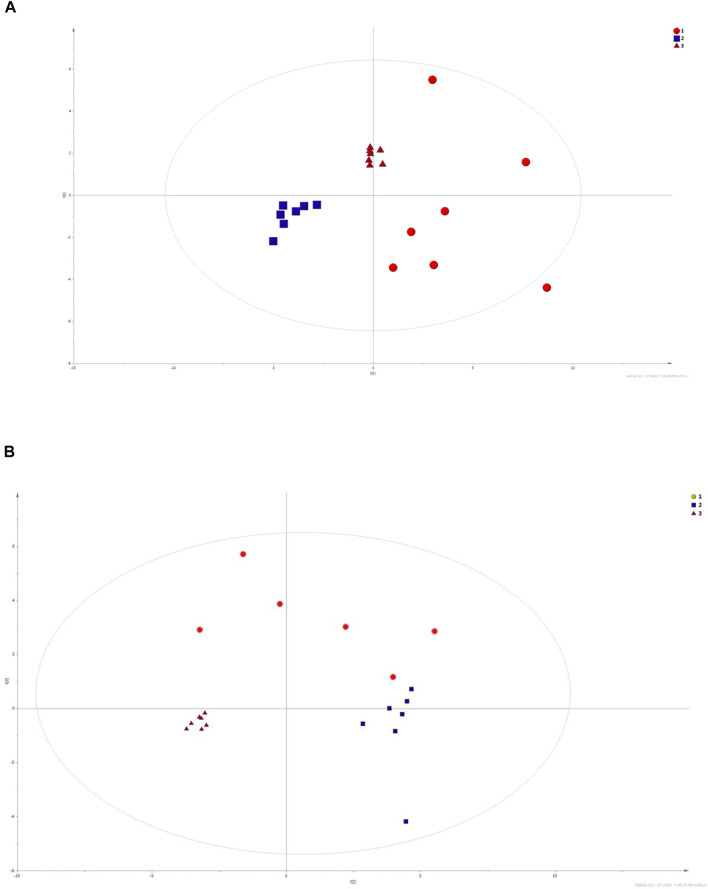
PCA models built on data obtained with LC-ESI-TOF/MS in positive mode and GC-EI-QqQ/MS technique. The calculated *R* ^2^ factors were *R* ^2^ = 0.725 and *R* ^2^ = 0.736 for LC-MS in positive ionization mode and GC–MS, respectively. Ejaculate samples are assigned by red circles (1). Blue squares (2) correspond to urine samples, while serum samples (3) are marked with red triangles.

PCA models were built based on the signals selected in accordance with quality assurance criteria. PCA is an unsupervised statistical procedure used to visualize general trends in obtained data and to compare three analytical matrices. PCA model is presented on the Figure 6. For this purpose, metabolites determined in all three matrices were selected. As can be observed, three separate clusters are displayed; triangles correspond to serum samples, and urine samples are represented by boxes, and circles reflect the ejaculate samples. Each of them corresponds to a different biological matrix. This may mean that each of the matrices has its own characteristic changes in molecular profiles, which allows for the determination of specific groups of compounds. The analysis of serum samples enables the determination of a wide spectrum of compounds with different physicochemical properties. Ejaculate samples are rich in fatty compounds, sterols, and sugars, while polar compounds are characteristic for urine samples.

Additionally, ejaculate samples are scattered which enables us to assume that metabolomics profiles of ejaculate samples are varied.

### 4.2 Univariate Statistics

#### 4.2.1 LC-ESI-TOF/MS analysis

Univariate statistics was performed in order to compare the abundance of a panel of metabolites determined in all three analyzed matrices. The application of ANOVA enabled to select 16 out of 34 variables, with an FDR value >0.05. Then, a *post hoc* Fisher test was used to explain which variable differs statistically significantly. The application of the *post hoc* test provides information about the differences in the three tested matrices, namely, ejaculate (1), urine (2), and serum (3).

The results are presented in the form of box plots with the use of two approaches. The first one is based on raw data, while the second corresponds to data after logarithmic transformation and autoscaling. This was to present data in a more clear way and to highlight the differences in abundances for the three analyzed matrices. All of the selected variables are presented in Table 3.

**TABLE 3:** Statistically significant variables for all three tested matrices determined with the use of HPLC-TOF/MS.

No.	m/z	Compound	Formula	t_R_	p-value	FDR	Post-hoc tests	Box Plot
*1*	162.1045	4,10-undecadiynal	C11H14O	21.305	0.011475	0.018032	1 – 2, 3 - 2	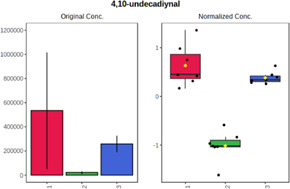
*2*	176.0474	10-Hydroxydecadienediynoic acid/10-hydroxydecenetriynoic acid	C10H8O3	23.613	1.73E-05	5.18E-05	1 - 2; 3 - 2	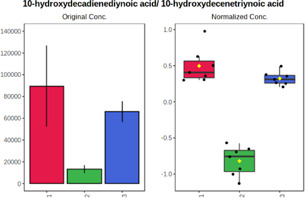
*3*	179.0584	Hippuric acid	C9H9NO3	13.44	2.76E-06	1.01E-05	2 - 1; 2 - 3	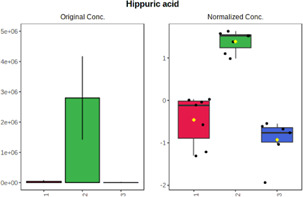
*4*	198.1617	Lauroleinic acid/lauroleic acid/	C12H22O2	25.21	3.19E-07	1.50E-06	1 - 2; 1 - 3	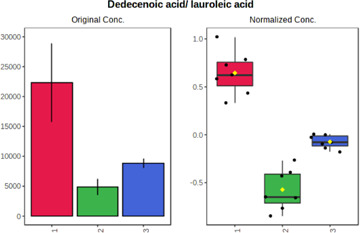
*5*	218.1876	8,8-Dimethoxy-2,6-dimethyl-2-octanol	C12H26O3	27.29	1.26E-07	6.94E-07	1 - 2; 1 - 3; 3 - 2	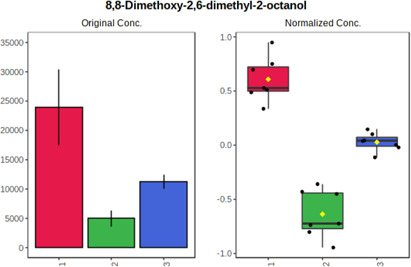
*7*	259.1782	Hexanoylcarnitine	C13H25NO4	13.70	0.016545	0.024818	1 - 2; 1 - 3	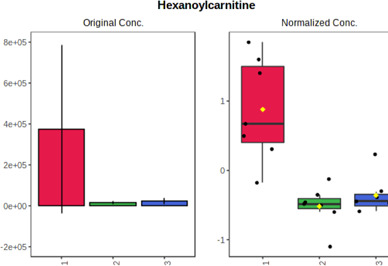
*8*	264.111	Hydroxymelatonin/	C13H16N2O4	13.07	1.54E-11	2.53E-10	2 - 1; 2 - 3	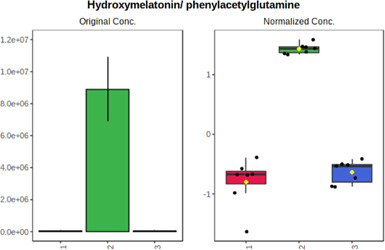
*9*	276.1726	Ginseoyne	C17H24O3	28.49	8.68E-05	2.20E-04	1 - 2; 1 - 3	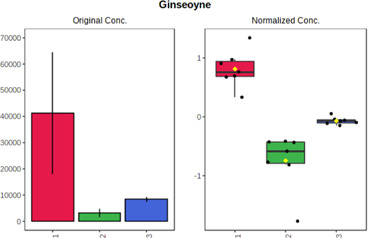
*10*	303.2043	3-Hydroxyoctanoyl carnitine	C15H29NO5	13.87	1.18E-05	3.89E-05	2 - 1; 2 - 3	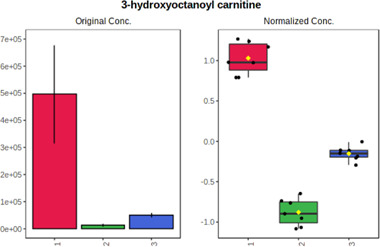
*11*	452.3363	Vitamin D3 derivative	C30H44O3	13.43	0.001281	0.002642	1 - 2; 1 - 3	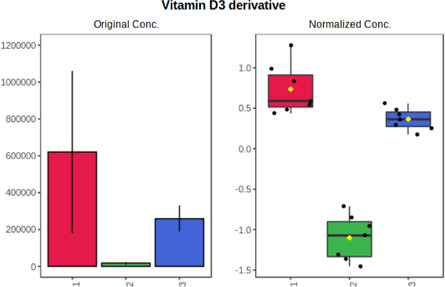
*12*	539.4025	LPS (19:0)	C25H50NO9P	14.41	0.01074	0.018032	1 - 2; 3 - 2	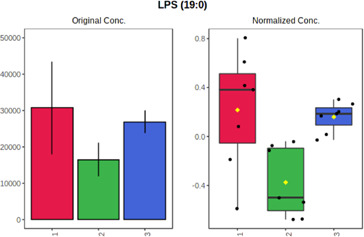
*13*	563.3513	PC (20:1)	C₂₈H₅₆NO₇P	13.00	0.001823	0.00354	1 - 2; 1 - 3	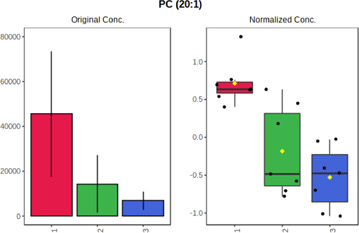
*14*	678.505	DG (40:8)	C44H70O5	14.21	7.16E-05	1.97E-04	1 - 2; 1 - 3; 3 - 2	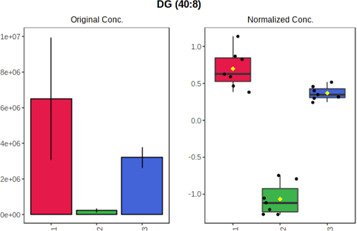

#### 4.2.2 GC–MS analysis.

In case of data obtained with the use GC-EI-QqQ/MS, the application of ANOVA enabled to select 24 out of 30 variables with an FDR value < 0.05. All of the selected variables are presented in Table 4. The application of *post hoc* test provides information about the differences in the three tested matrices, namely, ejaculate (1), urine (2), and serum (3).

**TABLE 4:** Statistically significant variables for all three tested matrices determined with the use of GC-QqQ/MS.

No.	Compound	tR	p-value	FDR	Fisher’s LSD	Plot
*1*	Hexadecanoic acid	21.71	1.7612e-08	4.5501e-07	1 – 2, 3 - 1, 3 - 2	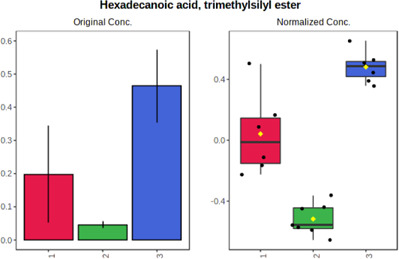
*2*	Arabitol	17.21	1.716e-11	8.8697E-11	1 – 3, 2 – 3, 2 - 1	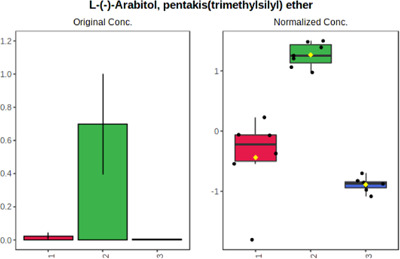
*3*	Octadecanoic acid	24.07	1.333e-11	8.2668E-11	1 – 2, 1 – 3, 3 - 2	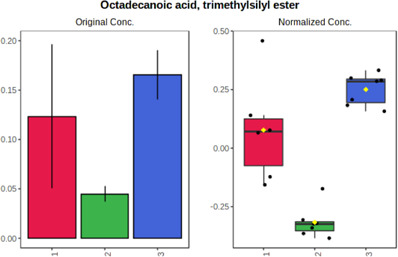
*4*	Threonic acid	14.56	2.0512E-9	7.0652E-9	2 – 1, 2 – 3, 1 - 3	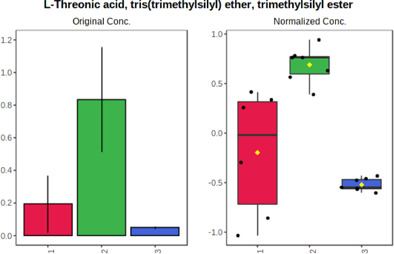
*5*	Threitol	13.96	1.6163E-6	2.1785E-6	2 – 3, 1 – 3, 2 - 1	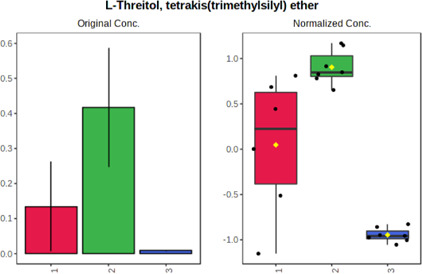
*6*	Fructose	19.37	2.5915E-6	3.3473E-6	1 – 3, 1 – 2, 2 - 3	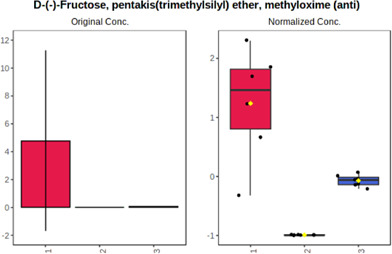
*7*	Lactic acid	6.15	6.4893E-7	9.1441E-7	3 - 2, 1 - 2	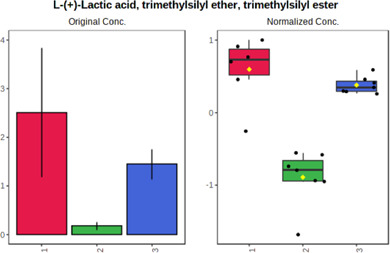
*8*	Propanoic acid	10.97	7.5801E-9	2.1871E-8	1 – 3, 2 - 3, 1 - 2	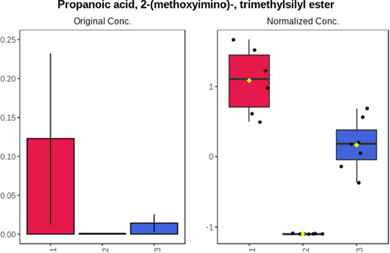
*9*	Talose	19.69	0.0045226	0.0048345	2 - 3	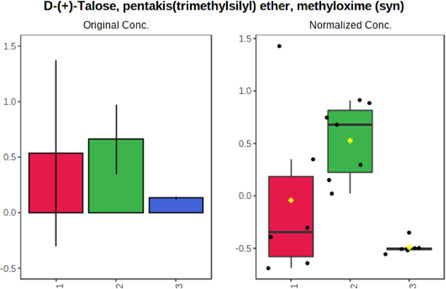
*10*	Myo-inositol	22.21	7.7605E-9	2.1871E-8	1 – 2, 1 – 3, 2 - 3	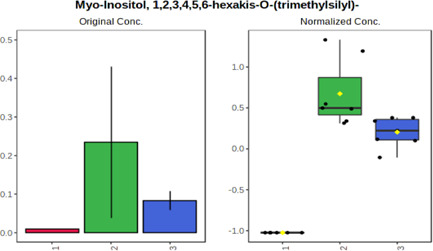
*11*	Dihydroxybutanoic acid	12.75	3.3783E-15	5.2364E-14	2 – 1, 3 – 1, 2 - 3	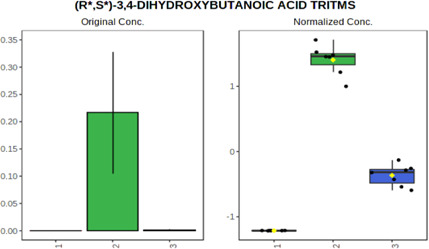
*12*	Galactose	20.00	4.6767E-14	4.8326E-13	2 - 1; 3 - 1; 3 - 2	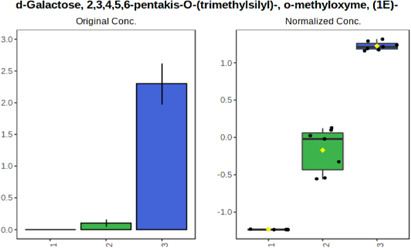
13	1,1,3-Propanetricarboxylic acid	18.66	4.5408E-12	3.5191E-11	2 - 1; 3 - 1; 2 - 3	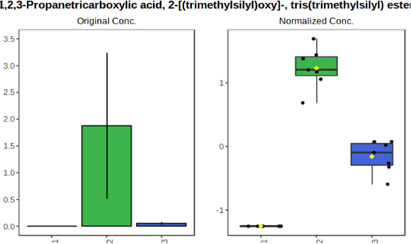
14	2-Methyl-3-hydroxybutyric acid	9.17	1.7124E-10	7.5833E-10	2 - 1; 3 - 1; 2 - 3	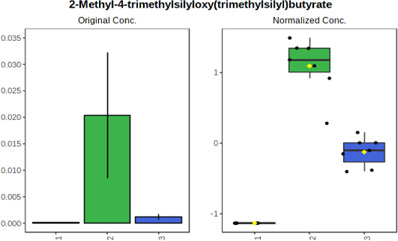
15	3-Hydroxybutyric acid	7.94	3.4838E-10	1.35E-9	1 - 2; 3 - 1; 3 - 2	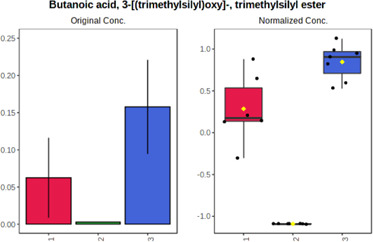
*16*	Octadecenoic acid	23.80	2.5732E-8	6.1362E-8	1 - 2; 3 - 1; 3 - 2	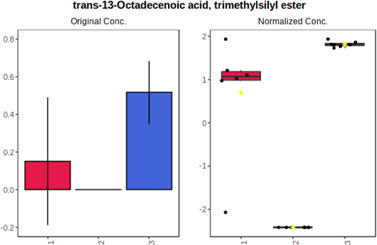
*17*	9,12-Octadecadienoic acid	23.72	3.412E-8	7.5552E-8	1 - 2; 3 - 1; 3 - 2	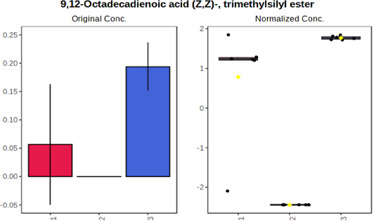
*18*	Cholesterol	33.04	1.9646E-7	3.3835E-7	1 - 2; 3 - 2	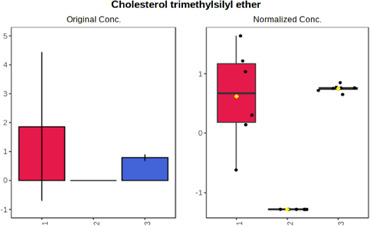
*19*	D-Sorbitol	19.14	3.8371E-7	5.9475E-7	1 - 3; 2 - 3	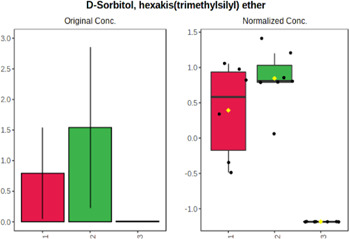
*20*	L-Isoleucine	10.34	6.8577E-8	1.3287E-7	1 - 2; 3 - 2	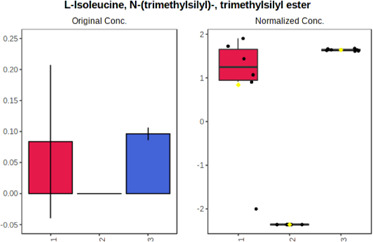
*21*	Glyceryl palmitate	27.72	0.021259	0.021259	1 - 2; 1 - 3	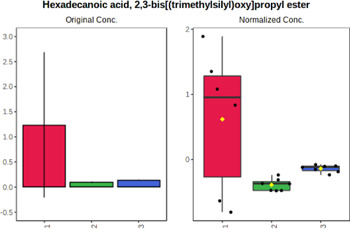
*22*	Glyceryl stearate	29.57	7.6307E-4	8.4483E-4	1 - 2; 1 - 3	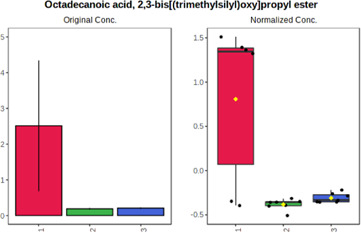
*23*	Glyceric acid	9.97	1.4525E-4	1.6677E-4	2 - 1; 2 - 3	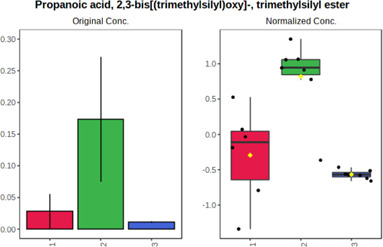
*24*	2-Hydroxybutyric acid	7.30	2.3645E-7	3.8579E-7	1 - 3; 2 - 3	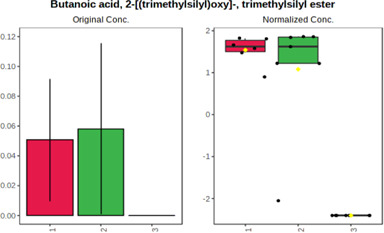

## 5 Discussion

Seminal fluid is a new, alternative matrix that may be utilized in metabolomics; however, due to ethical reasons, its application is not common. Metabolomics is one of the analytical tools which can be applied for seminal fluid mapping. Moreover, this approach may offer a new opportunity to evaluate specific factors of disease, such as potential biomarkers. The discovery of the metabolic signature of seminal fluid could lead to better understanding of the pathological mechanism of CaP. In practice, it may increase the diagnostic accuracy.

According to the literature, the composition of seminal fluid is very complex and contains a variety of molecules secreted by sex glands, spermatozoa (Murgia et al., 2020). The first part of our study was focused on the determination of the metabolic profile of ejaculate. However, due to the fact that the use of ejaculate in metabolomics studies is an undeniably novel development, the sample preparation protocol must have been prepared. The sample preparation step is crucial and analytically challenging. This step should include the extraction of metabolites which covers a wide spectrum of physicochemical properties. On the other hand, the preparation protocol should exclude interferences which lead to disturbance in the profile. It is even more important in the untargeted metabolomics approach where the interest is focused on the complex molecular signature of analyzed samples. Despite the development of numerous extraction techniques, liquid–liquid extraction (LLE) is still the most widely used method. By the selection of such parameters as the type or volume of the solvent, the process can be controlled and selective.

Because of limited studies regarding the metabolomics mapping of seminal fluid, three types of organic solvents were used: acetonitrile, methanol, and ethanol. This solvent selection was in accordance with the properties of solvents. Want et al. indicated that the procedure based on aforementioned organic solvents leads to obtaining the best metabolomics coverage (Want et al., 2006). Our results proved that the extraction protocol with 300 µL of methanol with 15-min incubation provided the highest number of metabolites with the highest reproducibility of analytical signals. Undoubtedly, the properties of methanol made it possible to obtain both hydrophobic and hydrophilic compounds. The extraction method using this solvent is widely applied in metabolomics. These results are in accordance with Want et al.’s evaluation of several extraction methods. The authors claimed that the application of organic solvent increases efficiency of metabolite extraction and protein precipitation in comparison to other methods like acid or heat treatment (Want et al., 2006).

Murgia et al. (2020) applied another type of LLE protocol for the determination of water-soluble metabolites in seminal fluid. For this purpose, methanol, water, and chloroform were used, but only the aqueous phase was analyzed with the use of the NMR-MS approach (Murgia et al., 2020). Seminal fluid was also applied for targeted profiling of steroids. Solid-phase extraction (SPE) was used by Olesti et al. (2020) for specific isolation of steroids from seminal fluid. The specificity and selectivity of the extraction process were evaluated in the study. Several parameters such as composition of washing condition and type of solvent mixture washed with water:methanol (95:5) were tested. The authors pointed out that elution with water:acetonitrile (10:90) and reconstitution with water:methanol (50:50) yielded the largest amount of steroids in human seminal fluid (Olesti et al., 2020). The choice of the extraction method and solvent was strictly dependent on the aim of the study. In our cases, complex mapping of seminal fluid was of interest. For this reason, methanol was selected as an extraction solvent. Its universal properties enabled the extraction of a wide range of compounds with different physicochemical properties. Metabolomics profiling enabled the acquisition of complex metabolomics signature of seminal fluid. Finally, 124 compounds were identified in the methanolic extract of seminal fluid using LC-ESI-TOF/MS. The metabolites were divided into chemical classes of compounds, where phospholipids, carnitines, amino acids, fatty acids, sterols, sphingosines, and glycerides were identified. Moreover, the application of the second technique enabled the extension of the profile with another 37 metabolites determined with the use of the GC-EI-QQQ/MS approach. This method enabled determining fatty acids, amino acids, one sterol and sugars, and their derivatives. Murgia et al. (2020) applied the procedure based on the Bligh and Dyer extraction protocol. This biphasic method is recommended for lipidomic analysis (Vale et al., 2019). Surprisingly, the authors used this protocol not for the lipidomic study but for the determination of water-soluble compounds. Similarly to our study, Murgia et al. (2020) identified amino acids, sugars, and their derivatives in seminal fluid.

Our study indicates that ejaculate can be a new alternative and fully informative matrix for the metabolomics study. Its analysis enabled determining the composition of ejaculate samples. According to the fact that erectile dysfunction appears in many elderly patients with CaP, the amount of the detected metabolites should be normalized when statistical evaluation is performed for comparison with non-cancer controls. The application of normalization by the total protein content or by wet weight measured in each seminal sample seems to be an adequate procedure to minimize the differences in erectile function among men. The application of two comprehensive analytical approaches led to determine few groups of compounds with varied physicochemical properties. Furthermore, the metabolic profile of the three tested matrices was compared in order to verify the hypothesis of the increased level of metabolites in ejaculate samples, related to the CaP development. For this purpose, metabolites present in all the three analyzed types of matrix were selected. Comparison of the individual metabolite levels shows a high similarity between ejaculate and plasma profiles. In both cases, the major composition is based on fatty acids, amino acids, sugars and their derivatives, phospholipids, carnitines, and sphingosines. The application of the two complementary analytical techniques enabled to obtain a complex metabolic profile. However, it should be pointed out that an increased level of specific groups was determined in serum samples, rather than in ejaculate. The increased level of phospholipids and sphinganine in ejaculate samples, rather than in serum, could also be observed.

The fingerprint of the ejaculate sample also converges to the metabolic profile of urine samples, especially in terms of polar compounds. However, similar to previous observation, levels of metabolites are increased in case of urine samples. Urine is a matrix which is characterized by a unique metabolic signature composed mostly of more polar metabolites. For this reason, the highest levels of organic acids and carnitines were reported.

In both cases, differences are significant and may not confirm the hypothesis of increased concentrations of individual groups of metabolites related to CaP in the ejaculate. On the other hand, it should be pointed out that ejaculate consists of mostly fatty compounds. Untargeted analyses and applied analytical parameters were provided in order to determine a wide spectrum of compounds. Obtained profiles are therefore only qualitative and not quantitative, so the comparison of levels of determined metabolites is only approximate.

Undoubtedly, it can be stated that the ejaculate is a matrix comprising the feature of both urine and plasma.

This discovery may lead to the wide use of seminal fluid in the diagnostics of urogenital diseases in the future. Based on the obtained results, both carnitine and lauroleic acid seem to be metabolites with potential diagnostic properties. However, it is worth noting that our studies are pilot, and validation on a larger set of samples is necessary. Moreover, the analysis should be also carried out in the negative ionization mode. Its application will allow for the determination of the lipidomic signature of semen samples. In particular, knowledge about the composition of fatty acids may validate the diagnostic potential of this matrix. To verify the hypothesis of higher concentrations of metabolites associated with CaP in the ejaculate than other matrices, extended research should be carried out. The results should be posted with age- and BMI-matched control group composed of healthy volunteers. Nevertheless, the following studies have proven that the ejaculate is an attractive matrix with high diagnostic potential.

## 6 Conclusion

In this study, a new and rapid extraction protocol from seminal fluid has been demonstrated. Three methods for metabolite extraction were compared; however, the application of methanol presents great yield in terms of the obtained metabolic profiles. Additionally, an optimized method was applied for untargeted metabolomics analysis of seminal fluid samples collected from CaP patients. This analysis enables to determine few classes of compounds with different physicochemical properties. The major one is fatty acids, sugars and their derivatives, amino acids, phospholipids, and carnitines. Finally, the metabolomics approach was applied to three matrices, urine serum, and seminal fluid samples, collected from CaP patients. The obtained metabolic profiles were compared in order to select these metabolites which were present in all three tested matrices. The results showed that ejaculate comprised specific features of both urine and plasma. Its potential to be a novel diagnostic matrix for urogenital diseases was proven.

## Data Availability

The original contributions presented in the study are included in the article/Supplementary Material, further inquiries can be directed to the corresponding author.
